# Performance of the Gail and Tyrer-Cuzick breast cancer risk assessment models in women screened in a primary care setting with the FHS-7 questionnaire

**DOI:** 10.1590/1678-4685-GMB-2018-0110

**Published:** 2019-06-03

**Authors:** Fernanda Sales Luiz Vianna, Juliana Giacomazzi, Cristina Brinckmann Oliveira, Luciana Neves Nunes, Maira Caleffi, Patricia Ashton-Prolla, Suzi Alves Camey

**Affiliations:** 1 Programa de Pós-Graduação em Epidemiologia, Universidade Federal do Rio Grande do Sul, Porto Alegre, RS, Brazil; 2 Programa de Pós-Graduação em Genética e Biologia Molecular, Universidade Federal do Rio Grande do Sul, Porto Alegre, RS, Brazil; 3 Laboratório de Medicina Genômica, Centro de Pesquisa Experimental do Hospital de Clínicas de Porto Alegre, Porto Alegre, Brazil; 4 Hospital Tacchini, Bento Gonçalves, RS, Brazil; 5 Serviço de Genética Médica, Hospital de Clínicas de Porto Alegre, Porto Alegre, RS, Brazil; 6 Associação Hospitalar Moinhos de Vento, Porto Alegre, RS, Brazil

**Keywords:** Breast neoplasms, risk assessments, family medical history, hereditary breast and ovarian cancer

## Abstract

Breast cancer (BC) risk assessment models base their estimations on different aspects of a woman’s personal and familial history. The Gail and Tyrer–Cuzick models are the most commonly used, and BC risks assigned by them vary considerably especially concerning familial history. In this study, our aim was to compare the Gail and Tyrer-Cuzick models after initial screening for familial history of cancer in primary care using the FHS-7 questionnaire. We compared 846 unrelated women with at least one positive answer to any of the seven FHS-7 questions (positive group) and 892 unrelated women that answered negatively (negative group). Concordance between BC risk estimates was compared by Bland-Altman graphics. Mean BC risk estimates were higher using the Tyrer-Cuzick Model in women from the positive group, while women from the negative group had higher BC risk estimates using the Gail model. With increasing estimates, discordance also increased, mainly in the FHS-7 positive group. Our results show that in women with a familial history of cancer, the Gail model underestimates risk and the Tyrer-Cuzick seems to be more appropriate. FHS-7 can be a useful tool for the identification of women with higher breast cancer risks in the primary care setting.

A wide variety of empiric and mathematical risk assessment models based on personal and familial risk factors have been developed to estimate a woman’s risk of developing breast cancer. Established risk assessment models to quantify breast cancer risk include the Breast Cancer Risk Assessment Tool (BCRAT), Tyrer–Cuzick (also called International Breast Cancer Intervention Study, IBIS), Claus and Ford models, BOADICEA and BRCAPRO, among others. However, these models base their respective risk estimations on different aspects of a woman’s personal and familial history and thus, are nopt equally well calibrated for all populations (Gail *et al.*, 1989; Claus *et al.*, 1991; Ford *et al.*, 1994; Antoniou *et al.*, 2004; Tyrer *et al.*, 2004; Amir *et al.*, 2010; Quante *et al.*, 2012).

The Gail and Tyrer–Cuzick models are widely used in several countries, especially in the United States, and currently are the only ones that incorporate both genetic and nongenetic factors. The utility of these models is to guide clinicians in decisions regarding age of initiation and periodicity of surveillance, need for genetic testing, and need to discuss additional risk-reducing interventions (Domchek *et al.*, 2003; Antoniou and Easton, 2006; Pruthi *et al.*, 2015). However, it is well known that the short-term and lifetime breast cancer risks assigned to a woman by the Gail and Tyrer–Cuzick models vary considerably. These differences are especially concerning family history (Amir *et al.*, 2010; Quante *et al.*, 2012).

Although family history of cancer is one of the most important tools for the initial identification of very high risk for breast cancer, little attention and time are usually spent to obtain a detailed pedigree in routine clinical practice. However, it is possible to perform accurate screening through simple family history questionnaires, even in the primary health care setting, as shown by our group and others (Ashton-Prolla *et al.*, 2009; Moyer and US Preeventive Task Force Force, 2014).

In the present study, we aim to compare the performance of the Gail and Tyrer-Cuzick models, and assess their concordance in women with and without a positive family history, as assessed by a questionnaire developed to identify high risk patients for hereditary cancer in the primary care setting. Breast cancer-unaffected women were recruited between March 2004 and February 2006 from a population-based breast cancer screening cohort in Brazil (Núcleo Mama Porto Alegre Cohort), and at inclusion in the study, all women answered the FHS-7 questionnaire about their family history of cancer (Ashton-Prolla *et al.*, 2009). Briefly, this questionnaire inquires about a history of: (a) breast (BC) or ovarian (OvCa) cancer in first-degree relatives, (b) bilateral BC, (c) male BC, (d) presence of BC and OvCa in the same relative, (e) BC before the age of 50 years, (f) presence of two or more relatives with BrCa and/or OvCa, and (g) presence of two or more relatives with BC and/or colorectal cancer. The patients that answered positively to at least one of the seven questions of the instrument were referred for genetic cancer risk assessment, which included comprehensive medical and family histories recorded in detailed pedigrees.

Two groups were included in the present study: (a) FHS-7 positive: 846 unrelated women with a positive family history of cancer (at least one positive answer to any of the seven questions), and (b) FHS-7 negative: 892 unrelated women that answered negatively to all questions of the same instrument. The study was approved by the Institutional Review Boards of the participating institutions.

The Gail breast cancer risk assessment tool (BCRAT) was constructed to estimate the risk of developing breast cancer in women undergoing annual mammographic screening. Briefly, it is an unconditional logistic regression model that provides a ratio of risk in women with specified risk factors compared with the risk in women without risk factors for the disease. The model is able to estimate current (within 5 years) and lifetime (up to the age of 90 years) risk of breast cancer (Gail *et al.*, 1989, 1999). Although it includes major risk factors for the disease, there are important limitations regarding family history of breast cancer and other tumors. The model considers only first-degree family history of the disease and does not include paternal history of breast cancer or male breast cancer, history of ovarian cancer and age at cancer diagnosis. The Tyrer-Cuzick Model was developed with data from the International Breast Cancer Intervention Study (IBIS) including a cohort of daughters of patients diagnosed with the disease. The input for the development was the estimation of probability of carrying a *BRCA1* or *BRCA2* mutation, as well as the estimation of breast cancer lifetime risks, through the analysis of family history, hormonal and reproductive factors, and personal characteristics (Tyrer *et al.*, 2004).

Breast cancer risk estimates (lifetime and within 5 years for the Gail model and 10 years for the Tyrer-Cuzick model), as well as demographic and clinical information were obtained in patient interviews. Since the upper age limit used in the two models differs (80 years in the Tyrer-Cuzick Model and 90 years in the Gail model), we modified the age limit of the Gail Model to age 80 years using the software BCPCARE kindly provided by Prof. Mitchell Gail to facilitate comparisons. To compare the risk estimates obtained with both models, the Bland-Altman method (Altman and Bland, 1983) was used, which is a graphical method to evaluate the agreement between quantitative measurements. The differences between the estimates were plotted against the average of the estimates, as well as bias and agreement intervals between the measurements. All analyses were done with R software (https://www.r-project.org/).

Table 1 summarizes the clinical and demographic data of women recruited for the study. The mean breast cancer risk estimates were higher when using the Tyrer-Cuzick Model in women from the FHS-7 positive group. The reverse was observed in FHS-7 negative women, who consistently had higher breast cancer risk estimates using the Gail model. In women with lifetime breast cancer risk estimates between 10 and 20% and in those with estimates > 20%, the Tyrer-Cuzick model always gave higher estimates than the Gail model. When comparing the estimates obtained with the two models, Bland-Altman graphs (Figure 1) show that in women with low breast cancer risk estimates (< 10%), the models are concordant, independent of the study group (FHS-7 positive or FHS-7 negative). However, as estimates increase, discordance also increases, and this is most evident in the FHS-7 positive group. Overall, in the FHS-7 positive group, the models show higher discordance in their estimates, in agreement with increased amplitude of the agreement interval (-8.76; 6.89) (Figure 1a). In the FHS-7 negative group estimates obtained from both models are more concordant, as can be concluded from the amplitude of the agreement interval (-2.75; 3.65). However, when analysing FHS-7 negative women with an estimated risk above 10%, the Gail Model underestimates the risk (Figure 1b).

**Figure 1:**
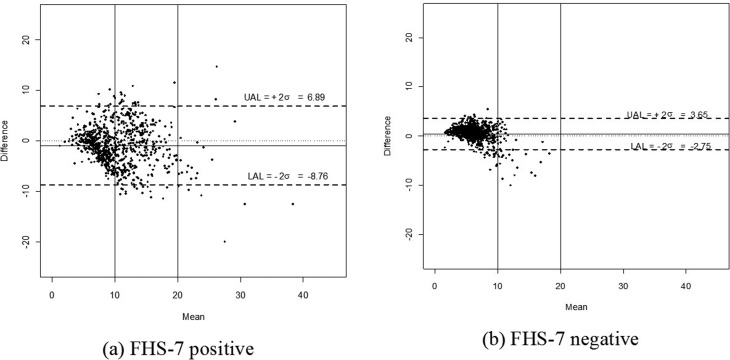
Bland-Altman graphics for the lifetime (up to age 80 years) breast cancer risk estimates obtained with the Models in the (a) FHS-7 positive and (b) FHS-7 negative. Mean: Average of Gail and Tyrer-Cuzick risks estimates. Difference: Difference between Gail and Tyrer-Cuzick risks estimates. UAL: Upper agreement limit. LAL: Lower agreement limit. FHS-7: familial history questionnaire.

**Table 1:** Descriptive data from demographics, reproductive and lifetime (up to age 80 years) breast cancer risk estimates variables in the FHS-7 positive and FHS-7 negative groups.

	FHS-7 positive (n=846)		FHS-7 negative (n=892)	
	N (%)	Mean (SD)	N (%)	Mean (SD)
Age at assessment	-	43.5 (12.2)	-	50.9 (9.1)
BMI	-	28.0 (5.6)	-	28.7 (5.8)
Age at menarche	-	12.7 (1.7)	-	13.0 (1.7)
Parity				
One or more children	753 (89.0)	-	828 (92.8)	-
Age at birth of first child	-	21.5 (5.0)		21.7 (5.2)
Reproductive Status				
Post-menopausal	295 (34.9)	-	449 (50.3)	-
Age at menopause	-	46.9 (5.5)	-	46.8 (5.5)
Hormone replacement therapy	69 (8.2)	-	97 (10.9)	-
Endogenous hormone exposure (ys)	-	3.3 (3.1)	-	3.0 (3.7)
Consanguinity^+^	58 (7.0)	-	52 (5.8)	-
Gail		9,9 (4,4)		6,5 (2,0)
< 10%	524 (61,9)		846 (94,8)	
10% - 20%	309 (36,5)		46 (5,2)	
> 20%	13 (1,5)		0 (0,0)	
Tyrer-Cuzick		10,8 (4,9)		6,0 (2,6)
< 10%	411 (48,7)		935 (93,6)	
10% - 20%	394 (46,7)		55 (6,2)	
> 20%	39 (4,6)		2 (0,2)	

(+) Evidence of consanguinity within family, regardless of relationship to the proband. BMI: Body mass index. SD: Standard deviation.

Lifetime breast cancer risk estimation using risk prediction models is an important step in defining breast cancer screening plans and breast cancer risk reducing interventions. Different risk estimation models have been published, among which the most commonly used are the Gail and Tyrer-Cuzick models. Previous studies have shown that these models perform differently in distinct scenarios and should be considered with caution for use in women at different risk levels (Amir *et al.*, 2010; Quante *et al.*, 2012) and National Comprehensive Cancer Network (NCCN) Guidelines 1.2015, Breast Cancer Screening and Diagnosis. Breast cancer risk assessment is particularly important in the primary care setting, but an important piece of information, the family history of cancer, is often overlooked. Thus, in this setting, obtaining a comprehensive pedigree is not a common practice, since it requires a significant amount of time and specific training. In the present study, we assessed the estimated lifetime risk of breast cancer obtained in a group of women participating in a breast cancer screening program in a primary care setting using the Gail and Tyrer-Cuzick models. All participants had been previously classified into two groups, as with and without a family history suggestive of high risk/hereditary cancer according to the FHS-7 questionnaire whose use is very straightforward. FHS7 is a simple questionnaire about cancer family history and although it does not take into account the full pedigree, and breast or reproductive/hormonal risk factors, it is fast and easy to use, ideal for initial risk screening in a primary care setting. We observed that in women responding positively to FHS-7, the discordance of the Gail and Tyrer-Cuzick estimates was higher, and in this group, the Gail model often underestimates the risk. Underestimation is directly related to the magnitude of risk.

Underestimation of lifetime breast cancer risk has relevant implications for a woman, since classification in one of the higher risk groups prompts the health care professional to discuss and/or recommend specific cancer risk reducing interventions that would not be offered just to any individual. These interventions include screening with mammograms and breast MRI, chemoprevention, and risk reducing surgeries for women carrying mutations in high penetrance cancer predisposition genes (Fisher *et al.*, 1998; Hartmann *et al.*, 1999; Rebbeck *et al.*, 1999; Moyer and US Preventive Task Force Force, 2014; Leach *et al.*, 2005; Lehman *et al.*, 2005;) and NCCN Guidelines 2.2015, Breast Cancer Risk Reduction.

For these reasons, breast cancer risk estimation is very important, and choosing a model that underestimates this risk may result in increased morbidity and mortality by breast cancer. At the same time, superestimation of risk may result in unnecessary screening procedures that will impose a burden to the patient and the health care system, which is especially relevant when one considers the high prevalence of breast cancer in different regions of the world and the increasing incidence rates observed in the past few years in several countries (Pollán *et al.*, 2009; Johnson *et al.*, 2013; Tassanasunthornwong *et al.*, 2015; DeSantis *et al.*, 2015).

Previous studies showed that the Tyrer-Cuzick is more accurate than the Gail model in certain setttings (Amir *et al.*, 2003, 2010; Jacobi *et al.*, 2009; Quante *et al.*, 2012). Our study agrees with the one by Jacobi *et al.* (2009) who compared results from different breast cancer risk estimation models in distinct risk scenarios, including presence or absence of a cancer history in the family. In their study, which involved examples of healthy counselees with different risk factors included in different pedigrees, they observed that in women without a significant family history of cancer, lifetime risk estimates varied from 6,7% (using the Gail model) to 12,8% (using the Tyrer-Cuzick model). However, when personal risk factors were included (especially family history), they concluded that the Tyrer–Cuzick model estimated the risk more accurately (Jacobi *et al.*, 2009). Our results are also in agreement with Quante *et al.* (2012), which compared the performance of these two models in subgroups of women without a strong family history and *BRCA*1 or *BRCA*2 mutation-negative versus subgroups typically classified as high-risk. The authors observed better overall calibration and discrimination for the Tyrer-Cuzick than for the Gail model in these high-risk groups (Quante *et al.*, 2012).

Therefore, several authors have shown that the Tyrer-Cuzick model performs accurately in the identification of moderate to high risk of developing BC, and currently it is the instrument that includes the largest number of established BC risk factors (Amir *et al.*, 2010). However, it requires more time of interview with the patient compared to the Gail model and also more information, especially on family history of cancer. Thus, FHS-7 could be used as an initial cancer risk assessment tool to screen for higher risk cases, that could then be submitted to the Tyrer-Cuzick model evaluation (Figure 2). The approach through FHS-7, is straightforward and fast, does not require extensive training, and could be especially useful in screening large populations.

**Figure 2:**
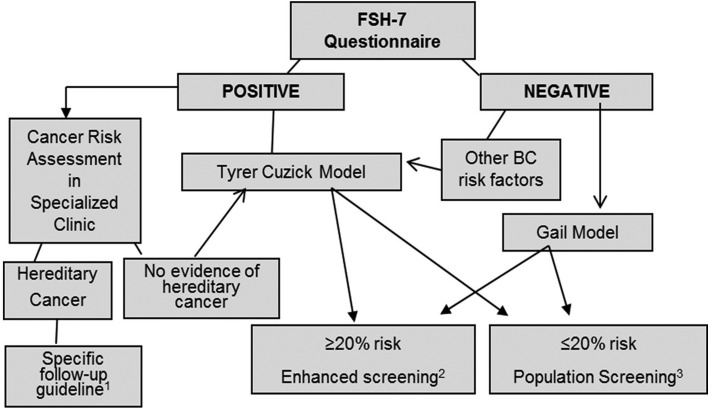
A strategy for breast cancer risk assessment in primary care based on family history screening. 1: As suggested by the National Comprehensive Cancer Network (NCCN) Guidelines 1.2015, Breast Cancer Screening and Diagnosis (NCCN, 2015). 2: As suggested by Saslow *et al.* (2007), Pruthi *et al.* (2015), and NCCN, 2015. 3: According to local policies for breast cancer population screening.

In conclusion, we show that, after an initial screening for family history of cancer using a simple questionnaire, the Gail and Tyrer-Cuzick models show different performance profiles according to the presence or absence of specific high-risk family history features. We observed that both the Gail and Tyrer-Cuzick models perform similarly in individuals without a significant cancer family history, but in those with high-risk features from the family history of cancer, or from reproductive and/or hormonal risk factors, the Gail model underestimates risk. Potential limitations of this study include the low specificity of FHS-7 and the absence of long-term follow-up regarding breast cancer diagnosis as an outcome in both study groups. However, our proposal was to use FHS-7 only as an initial family-history screening tool in the primary care setting, and thus qualify the choice of a breast cancer risk assessment strategy that is most appropriate for each clinical scenario. The assessment of familial risk of breast cancer in a primary care setting is particularly relevant in populations with high incidence rates of the disease.
